# Whose Rights Count? Negotiating Practice, Policy, and Legal Dilemmas Regarding Infant–Parent Contact When Infants are in Out-of-Home Care

**DOI:** 10.1002/imhj.21381

**Published:** 2013-02-19

**Authors:** Devi Miron, Claud Bisaillon, Brigid Jordan, Graham Bryce, Yvon Gauthier, Martin St-Andre, Helen Minnis

**Affiliations:** Tulane University School of MedicineLouisiana, USA; Université de SherbrookeQuebec, Canada; Murdoch Childrens Research Institute, Royal Children’s HospitalParkville, Victoria, Australia; National Society for Prevention of Cruelty to ChildrenGlasgow, Scotland; Université de MontrealQuebec, Canada; University of Glasgow, Royal Hospital for Sick ChildrenGlasgow, Scotland

## Abstract

This article takes a human rights perspective with a view to articulating the infant’s perspective when the infant has been subjected to abuse, neglect, or both and is reliant on the state to ensure his or her health and well-being. When a young child is removed from parental care, important and often difficult decisions have to be made about subsequent contact between child and parent. We consider a number of dilemmas which may arise for practitioners when they are assisting child welfare decision makers in relation to contact, and acknowledge the limited empirical follow-up studies of the impact of child welfare practice and legal decisions on infant outcomes. We draw on the significant and substantive evidence base about infant emotional and cognitive development and infant–parent attachment relationships as well as infant mental health to illuminate the infant’s subjective experience in these practice dilemmas. We describe innovations in practice from various countries, which seek to shed light on the challenges often associated with contact.

Some of the most challenging issues in child welfare practice arise in the dilemma of ensuring that an infant removed from his or her parent’s care is provided with a secure attachment and caregiving relationship with someone who is not their biological parent while also working to ensure that the best possible circumstances are provided for the infant to have an ongoing meaningful relationship or to be united with their parents when this is in the infant’s best interests. One of the most contested issues internationally is how frequent contact with parents should be for very young infants in out-of-home care. A simplistic rendition of attachment theory is often used to justify arrangements that are extremely onerous for infants, such as visiting 7 days a week with biological parents. Infant mental health practitioners are often called upon for consultation on these difficult decisions. Practitioners may be asked to evaluate children and families for the child welfare system or for the courts and to render an opinion about the best interests of a child who is residing in foster care. There is sparse empirical or clinical literature to guide practitioners in conducting such evaluations and assisting with decision making.

In this article, we offer insights about visitation between parents and their young children who are residing in foster care due to maltreatment or severe neglect. We draw from our experiences in consulting to the child welfare systems in four nations \Australia, Canada (Province of Quebec), Scotland, and the United States]. While there are certainly differences, we have observed many similar challenges across these developed countries, which have the intention and resources capable of serving the best interests of maltreated children. All of these countries have overloaded foster care systems, with a substantial proportion of children in foster care under the age of 5 years. Further, the number of children under the age of 5 years in care has increased dramatically over the last decade (Adoption and Foster Care Analysis and Reporting System \AFCARS], 2009; Australian Institute of Health and Welfare \AIHW], 2009; The Scottish Government, 2011).

We use the human rights perspective employed by our Australian colleagues as a framework for viewing practice dilemmas in child welfare. A brief review of the needs of infants in out-of-home care is provided first. We then consider parent–child contact as the context in which many policies and practices intersect and where, too often, the needs and best interests of young, traumatized children in care, including safety; physical, developmental and psychological well-being; attachment security; stability; and permanency planning, are not being adequately addressed. We also discuss the challenges of educating and consulting to child welfare staff and the courts^1^ about basic infant mental health concepts to consider when making decisions regarding the amount of contact infants have with their birth parents. We report case studies from Canada, Scotland, and the United States to illustrate the challenges faced by clinicians, mental health programs serving young children in foster care, the child welfare systems, and the courts when prioritizing the needs of infants while evaluating the merits of trying to reunify the particular family. Throughout the article, we make recommendations for infant mental health practitioners who consult to child welfare staff about structuring and facilitating high-quality visits so that the visits may act as evaluation tools in making case plan decisions and, when indicated, maintaining or establishing an optimal bond between infant and parent. We conclude with a discussion of what is not yet known about the experiences of young children in foster care.

## A HUMAN RIGHTS PERSPECTIVE: A VIEW FROM AUSTRALIA

In their Australian Institute of Family Studies article, Jordan and Sketchley (2009) considered the needs of young children in foster care from a human rights perspective. A human rights perspective views child maltreatment as a violation of the child’s rights. The United Nations Convention on the Rights of Children in 1989 described the civil, political, social, economic, and cultural rights of children. They include rights to participation and provision in addition to the right to protection. Thus, all children have the right to experience the conditions for optimal health, growth, and development and that society has an obligation to ensure that parents have the necessary resources to raise children (Reading et al., 2008). The guiding principles include, but are not limited to, the best interest of the child; the other principles are nondiscrimination, survival and development, and respect for the views of the child (Webb, Horrocks, Crowley, & Lessof, 2009). The articles of the convention are not abstract ideals but can be translated into specific interventions and policy objectives and inform ethical decision making in child welfare practice (Reading et al., 2008). They are legal obligations in signatory countries, and the idea of abuse as a violation of the child’s rights can strengthen the position of the infant in the context of societal ambivalence about ensuring that the interests of children take precedence over the ideology of the family or the rights of parents. This article explores this translation for infants in out-of-home care.

Jordan and Sketchley (2009) argued that a human rights perspective means that decision making for infants in out-of-home care should consider the subjective experience of infants. As cogently argued by Gauthier, Fortin, and Jeliu (2004), even if parents do overcome their difficulties, from a human rights perspective the best interests of the child means that reunification or visiting schedules should not be a “prize for parent’s rehabilitation.” This perspective can ensure infants’ needs are a priority in decision making when encountering child welfare practice dilemmas and when practitioners are asked to assist with resolving such dilemmas.

“Articulating the infant’s perspective” means communicating key information to the parties involved so that they can appreciate, for example, the extent to which babies are still developing the capacity for emotional regulation and self-soothing in the first few weeks and months of life, and are highly reliant on the sensitive and emotionally available presence of their caregiver (Brazelton & Cramer, 1990). Most can tolerate only brief periods of separation from their primary caregiver, who, for maltreated infants in care, is usually the foster parent. This is especially true if a baby’s capacity for self-regulation is compromised in any way (e.g., by being medically fragile, suffering withdrawal from opiates at birth, or having persistent crying or feeding difficulties). In these instances, the baby’s own body might be experienced as being unpredictable so that they are especially reliant on the continuous, predictable responses from their caregiver to be not so frightened and to develop confidence that they will get over their upset. These processes of interactive emotional regulation and the development of emotional security have often been severely disrupted by the neglect or maltreatment, which has led to the infant being placed in out-of-home care. It is critical that visiting schedules do not involve unmanageable separations from the foster caregiver that will undermine the developing relationship and cause emotional distress or further traumatise the infant. Maltreatment and the often repeated separations from caregivers mean that infants and toddlers who are placed in out-of-home care are at increased risk of mental health disorders.

## NEEDS OF INFANTS IN OUT-OF-HOME CARE

Little empirical research exists concerning the experiences of infants residing in foster care. The studies that do exist focus on needs such as attachment or developmental (e.g., Smyke, Zeanah, Fox, Nelson, & Guthrie, 2010) or behavioral and emotional symptomatology (Ghera et al., 2009; Robinson et al., 2009). A recent article in the *Journal of Child Psychology and Psychiatry* (*JCPP*) reviewed the emotional, behavioral, neurobiological, and social vulnerabilities of children in care as well as evidenced-based interventions that promote resilience (Leve et al., 2012). The authors discussed two important factors, placement disruption and prenatal exposure to substance abuse, which contribute to negative outcomes for children in care. However, there are few guidelines for how to use the existing research on the needs of young foster children to make decisions that optimize outcomes. On the other hand, a great deal of fundamental, scientifically established knowledge about infant development exists that can help guide decision making by infant mental health, child welfare, and legal practitioners. In this article, we illustrate how this knowledge can be applied by practitioners to understand the needs of young children in foster care and to assist child welfare workers and courts in making decisions specifically about contact with birth parents.

There is an abundance of research on attachment development that is certainly applicable when considering the needs of young children, but it is beyond the scope of this article to review this entire body of studies. On occasion, attachment theory is appropriately invoked by child welfare and court officials to explain infants’ needs for ongoing contact with their parents. However, Bowlby (1969) also drew attention to the way in which attachment behaviors and exploratory behaviors are complementary. If feeling overtaxed or stressed, attachment behaviors (clinging, crying) escalate, and exploratory behaviors shut down. From this perspective, the arrangements for infants to visit their parents need to ensure that the infants are feeling as secure as possible, alert, awake, and happy for them to have the curiosity and emotional energy to invest in getting to know and to interact with their parents. This is especially true if the parents have never been the primary caregiver for the infant.

In addition to attachment research, recent infant development discoveries shed light on the decision-making process regarding contact with birth parents for young children in out-of-home care. Over the last few decades, “still face” experiments and research on neonatal imitation have demonstrated infants’ capacity for primary intersubjectivity (awareness of the feelings and motives of others) and their distress when the “rules” of reciprocal communication have been broken. A birth parent’s attunement to the infant while visiting offers opportunity for promoting attachment and development. On the other hand, constant misreading of an infant’s cues and misattributions of an infant’s intent within an interaction are a significant source of stress for the infant (for case illustrations, see Ostler & Haight, #imhj21381-bib-0025^2011^).

Recent developments in neuroscience research, particularly the discovery of “mirror” neurons as the intention-detection center of our brains, highlight infants’ capacity to detect and be affected by the state of mind of the person with whom they are interacting (Meltzoff & Brooks, 2007; Stern, 2008). Moreover, developmental traumatology research has demonstrated that the architecture of the infant brain—and subsequently, cognitive and affective functioning—are affected by early life stress (Pechtel & Pizzagalli, 2011). Thus, being in the presence of an adult whose state of mind is very disturbed—whether hostile or harsh or belittling—risks having long-lasting impacts on a developing infant.

### The Context of Visitation

#### Purpose and prospective benefits of visits

In their review of the literature, Sen and Broadhurst (2011) evaluated the benefits of contact between children in care and their biological parents that have been claimed. They concluded that the correlations between contact and reunification and contact and placement stability are complicated by the variability of the circumstances of the child being in care. They also submitted that the impact of contact on developmental outcomes of children is not well-evidenced. They suggested that it is not contact alone but the combination of contact with other family services that improves chances for reunification. The authors cautioned against making broad prescriptions for all children, given that circumstances are highly individualized.

Clearly, the visiting needs of infants and young children residing in out-of-home care are different from those of older children and adolescents. The cognitive development of older children allows for a better understanding of the circumstances under which they see their parent. Smariga (2007) articulated the benefits of family visiting time for infants in out-of-home care and their parents (whose needs are often prioritized), and suggested that it

promotes healthy attachment and reduces negative impacts of separation for the child and parents, establishes and strengthens the parent–child relationship, eases the pain of separation and loss for the child and parent, keeps hope alive for the parents and enhances parents’ motivation to change, involves parents in their child’s everyday activities and keeps them abreast of the child’s development, helps parents gain confidence in their ability to care for their child and allows parents to learn and practice new skills, provides a setting for the caseworker or parenting coach to suggest how to improve parent–child interactions, allows foster parents to support birth parents and model positive parenting skills, provides information to the court on the family’s progress (or lack of progress) towards their goals, facilitates family assessments and can help the court determine whether reunification is the best permanency option for the child, helps with the transition to reunification. (p. 6)

*Visits as ongoing evaluation and intervention opportunities*. In some agencies or jurisdictions, child welfare workers use visits as opportunities to assess the parent–child relationship. These visits are typically scheduled regularly, with the understanding that the parent will address the circumstances that brought the child into care, such as mental health or substance abuse, between visits with his or her child. Unfortunately, visits often continue without the parents having made any meaningful progress on their individual issues and without intervention during the visits to support the child or the relationship. Because the infant–parent relationship is often unhealthy prior to placement, visits should be regarded as planned therapeutic intervention that offers an opportunity to heal a damaged relationship and therefore be resourced and evaluated with this framework in mind. The best outcome for infants would be ensured by visits being used as an opportunity to evaluate the strengths and concerns of the current relationship and, when indicated, being supported by skilled therapeutic intervention to heal the infant–parent relationship. Even when visits are intended for evaluation only, therapeutic intervention should be provided if it becomes clear that the infant is being harmed. This intervention may range from simple to sophisticated, depending on the needs of the dyad, and would offer relationship-building and emotional support for the child and parent during the visit. Ongoing assessment of progress and the demands on the infant would follow, given that the parent–child relationship is dynamic and evolving. As therapeutic visitation continues, reappraisal and reworking to a realistic and safe schedule should occur if the infant is distressed by the visits or the parent does not attend. Visit schedule and structure should be reviewed according to the infant’s developmental stage, emotional development, and the dynamics of the infant’s relationships with both the caregiving family and the parent. Infant mental health practitioners are well-informed to assist child welfare workers in considering the issues when planning and structuring contact.

### Issues to Consider When Planning and Structuring Visits

#### Quantity versus quality of contact

Any potential benefit of parent–child visiting will not automatically eventuate as a result of face-to-face contact alone. The infant’s subjective experience will depend on a myriad of factors such as whether parents were ever the primary caregivers for the infant, the quality of interactions prior to removal, the distress involved in separation from the foster parent for the duration of the visit, and the likelihood of the infant returning to live with the birth parents. An Australian study by Humphreys and Kiraly (2009), in one state, reviewed the case files of all infants in care who were under 1 year of age. Thirty-four percent of these infants had court-ordered visiting of four to seven visits per week, but this was only sustainable by parents in half the cases due to their life circumstances. This study found that high-frequency visiting schedules were not associated with increased rates of reunification with parents 1 year later.

The whole issue of quality of time versus frequency and length of time for visits needs careful thought. The optimal amount of contact after the initial transition to placement may well depend on what “costs” are involved in the visit. These include factors both *surrounding* the visit, such as separation from the new caregiver; lack of attachment figure during transport; and disruption to sleep, play, and other daily routines; and factors arising *during* the visit, such as misattuned or even distressing interactions with the parents and conflict between the parents. Many people have advocated for less frequent, longer blocks of time for the infant and parent to spend together, but the infant’s subjective experience of such arrangements depends on the age of the infant, the infant’s capacity for self-regulation, and the quality of interactions with the parents. A long block of time with parents means a longer block of time away from their secure base attachment figure (foster parent). Note that infants growing up in the community with their parents where there are no protective concerns, develop secure attachment relationships with grandparents over time on contacts as infrequent as once a week or less.

#### Suspension of visits

Literature that has argued for frequent contact usually has acknowledged that there may be circumstances where visits should be ceased, such as if there is a risk that parents will physically or psychologically harm the child or if the visits are extremely traumatic for the child (Goldsmith, Oppenheim, & Wanlass, 2004; Haight, Kagle, & Black, 2003; Smariga, 2007). When parents have assaulted an infant, the mere presence of the parent has the potential to be a traumatic reminder. Particular states of mind in an infant (e.g., fear or terror) can be encoded as an implicit form of memory, and these states can be reactivated in the presence of the abusive parent (Berger & Rigaud, 2001; Lieberman, 2004; Siegel, 2001). Specific aspects of the parent’s behavior, such as voice, body movement, or facial expression, can be a traumatic reminder that signals danger to the infant that an attack is imminent \Based on past experience, this is a realistic automatic appraisal by the infant (Lieberman, 2004).] In these circumstances, the infant’s subjective experience is of being reminded and terrified of the terror and pain of abuse. Such visits are likely to cause emotional suffering, hypervigilance, and effects similar to the impact of the original abuse. These dangers are heightened when the visits occur without the infant having his or her primary caregiving adult (foster parent or kinship caregiver) present. A human rights perspective suggests that visits with parents should provide a benefit *to the infant* and no physical or emotional costs or risks for the child. Visiting arrangements also need to ensure that the infant has the best chance of recovery from abuse and neglect. Finally, designing optimal visiting arrangements for infants who will never return to live with their parents is a complex issue. A rights-based perspective requires us to ensure that the rights of infants who have been harmed by abuse and neglect take precedence over parents’ rights.

### Current Practice and Challenges to Communicating with Child Welfare and Legal Systems

Haight, Kagle, and Black (2003) proposed several excellent attachment-informed recommendations for child welfare policy and practice around planning and supporting parent visitation with their infants in care. First and foremost, they proposed that adequate attachment relationships between children and their foster and biological parents should be supported. They suggested supporting regular and frequent visitation for young children with their biological parents in a socially and culturally appropriate setting *whenever reunification is a viable goal*. The authors advised supporting parents and children before, during, and after visits. In addition, given that the child’s primary attachment relationship may be with the foster parent due to the fact that she or he is providing the daily care to the child, they offered that foster parents play an important role in managing visits. To maintain this relationship, they suggested that social workers adequately prepare and support foster parents for providing corrective attachment experiences for children who need this. The authors acknowledged that there may be instances in which visits should be therapeutic, reduced, or suspended, for example, when a disorganized attachment relationship has been identified. Further, they suggested that priority should be placed on service plans for parents such as mental health or substance-abuse treatment and that visiting plans should be coordinated with progress in therapy.

Even with these recommendations published nearly a decade ago, we have found that such practices are very difficult to implement in many jurisdictions. In our experience, the consideration of infant needs in planning for visitation is often inadequate or completely disregarded in favor of agency, parent, or court priorities. Infant visits with parents may be scheduled around agency needs (e.g., availability of staff to transport infant, practicalities of infants visiting in the morning to ensure afternoon slots are available for school-aged children, etc.) rather than being determined by the individual daily timetable of the infant. This reduces the potential benefit of visits, as the infant is likely to have his or her own biological rhythms disturbed to be put in the car and thus is less likely to be in the alert and calm state that promotes quality interaction with and responsiveness to parents during the visit. For example, when an infant is woken from sleep to be taken to a visit, he or she is likely to arrive tired and cranky, may be fed whether a feed is due or not, and may become so overwhelmed that he or she falls asleep to cope with the visit, which is unsatisfying for both infant and parents. Further, caseworkers are often hesitant to include foster parents to support infants during visits between children with their birth parents because of the fear or uncertainty around managing the birth parent’s feelings about having time with their child “intruded upon” by the foster parent. Finally, suspension of visits may be indicated from the perspective of the child, but the approval of this may be delayed or rejected by the court system, which sometimes considers the rights of parents as paramount; consequently, children may be further harmed by continued contact with their parents. This is why “articulating the infant’s perspective” to all involved is so important.

Even though those involved are committed to helping children, we have found that communicating both the immediate and long-term needs of infants in foster care to child welfare workers and court officials is often challenging. Child welfare workers, on a daily basis, and the court, more globally, are in the position to either allow or prevent further harm to already vulnerable, traumatized infants. Workers often do not have the training necessary for assessing harm (especially emotional or psychological harm) from the perspective of the infant. In some parts of the United States, to become a child welfare worker requires simply a bachelor’s degree in any field. Even for those who have obtained a degree in health or welfare, many undergraduate programs do not focus on typical infant development. This is the case internationally. Thus, there are large gaps in knowledge and understanding of infant development and the infant-sensitive practice that is required to work with families and infants to ensure their safety and well-being (Jordan & Sketchley, 2009). In addition, while those who make the decisions in the legal forum are necessarily well-informed about the law relating to children, they seldom have basic understanding of infant development or infant mental health. This knowledge gap poses an extremely precarious situation from the vantage point of the infants residing in foster care, as their well-being depends on decisions made in the here and now by their workers and judges. Some innovative developments, such as the infant/toddler “court teams” (ZERO TO THREE, 2006) established in several cities around the United States have begun to address this gap, but such models are the exception rather than usual practice in most jurisdictions around the world.

## ADDRESSING CHALLENGES

The question is: How do infant mental health practitioners begin to ensure that child welfare and legal systems operate from the infant’s perspective? Put simply, infant mental health practitioners are well-placed to provide child welfare and legal practitioners with an understanding of infant development, including emotional development and infant mental health concepts, which should be considered when making decisions. The following sections expand on recommendations for practitioners, and case examples illustrate how effective practice from the infant’s perspective is being done in child welfare jurisdictions in developed nations around the world.

### Recommendations for Infant Mental Health Consultants to Child Welfare Systems on Issues of Contact

**1**. Infant mental health practitioners should offer training in infant mental health for child welfare workers and legal practitioners.

One major obstacle in practicing in the best interests of the child is that child welfare workers typically do not have the knowledge or skills to evaluate the nuances of infant–parent relationships or the skills to intervene therapeutically. More sophisticated therapeutic dyadic interventions would, of course, require advanced training in infant mental health. However, given the central role which child welfare workers play in the lives of these very vulnerable children, there may be a case for including basic principles of infant mental health in training courses for these workers. Further, legal practitioners must be educated on these issues. In fact, it may be up to the worker to communicate the issues to the legal system, for example, during court testimony. Thus, workers need to have the tools to relay the infant’s perspective to the court.

**2**. Infant mental health practitioners should offer consultation to the child welfare and legal systems around practical day-to-day and global decision making about contact with biological parents from a “children’s rights” perspective.

In addition to training, or perhaps when training is not possible, child welfare and legal practitioners would benefit from ongoing consultation from infant mental health specialists about the needs of young children residing in out-of-home care. For example, in jurisdictions where foster parents do not transport infants to or accompany infants during the visits with their parents, the infant’s subjective experience while being transported to and possibly during the visit can be an experience of emotional abandonment. This is not optimal for relationship-building, and if arrangements involve handling by multiple people within a day or over a week, then visits are likely to be highly stressful, counterproductive, and in fact damaging. Such decisions need to be made with the infant’s experience in mind. The cases presented next illustrate how such consultation has been implemented, has been received, and has affected decision-making in different international jurisdictions.

**3**. Infant mental health clinicians should offer direct services for children in out-of-home care and their caregivers to optimize contact.

Whenever possible, infant mental health clinicians should provide direct intervention concerning issues of contact when children are in out-of-home care. This may take place in the form of evaluating the quality of the child–parent relationship and the experience of the visits from the child’s perspective and then when necessary, providing therapeutic visitation services. The Tulane Infant Team (Zeanah et al., 2001) offers a model for global evaluation and focused intervention services for young children in care, and Marty Visit Coaching (Beyer, 2008) is an approach for optimizing contacts.

## INTERNATIONAL CASE STUDIES OF INFANTS IN OUT-OF-HOME CARE

Having discussed children’s rights in relation to parents’ rights, in this section, we focus on foster children’s reactions to court-ordered visits with their biological parents. We present clinical vignettes (de-identified) to describe such reactions as manifestations of severe stress on these children’s attachment systems and discuss recommendations to make such visits more “livable” for foster children and their families. We also describe efforts to contribute to the research base regarding the experiences of young children in foster care. Drawing from three countries, the scenarios described illustrate common practice dilemmas in the clinical, court, and child welfare arenas. The child welfare policies of these nations are orientated toward permanency planning for infants in out-of-home care. This often means concurrent planning for the reunification of the child with his or her biological parent as well as for adoption by a nonbiological parent. Therefore, the infant’s experience and the ability of all caregivers and service providers to meet the needs of the infant while in care are considered in the case descriptions.

### The Experience of the Child: Case Vignette from Quebec, Canada

#### Quebec judiciary context

The Quebec Youth Protection Law is oriented toward family preservation (Government of Quebec, 2006). Significant modifications introduced in 2006 include an obligation for permanency planning (e.g., placement until majority) and provide mandatory guidelines for temporary placement of children according to their age: Maximum durations vary from 12 months for children aged 0 to 2 years, to 18 months for 2- to 5-years-olds, and to 24 months for children 5 years and older. In line with the family preservation orientation, contacts between the child and his or her biological family are assumed to be beneficial. In the following vignette, we demonstrate how the effects of such contacts can have negative consequences for the child and how clinicians, the child welfare system, and the court must adjust their intervention in the interest of the child.

#### Background

Brandon was placed with his paternal grandmother at 3 months due to parental neglect. The Court had ordered a placement until his majority when he was 11 months old. When he was 30 months, his biological mother, whom he saw for 4 hr every week at her home, was granted a court request to increase her contacts to 3 days a week. Following this radical change of visitation rights, Brandon began showing symptomatic manifestations after most weekly visits: clinging behavior, aggressive outbursts, sleeping difficulties (up to four nocturnal awakenings where he cried for grandmother). The symptoms lasted from 2 to 3 days, and then remitted until the next visit. Despite those manifestations, visits stopped being supervised after 1 year. Brandon then started hiding from his mother when she arrived for the visit; moreover, Brandon presented selective mutism throughout the visits. Several months later, his mother revealed that she promised her son that he could come live with her. Brandon’s mother then started a new job and requested whole day visits, but only once a week.

#### Practice dilemmas

The issues for all involved with Brandon were threefold: (a) to determine the cause(s) of his symptoms; (b) to find ways to support Brandon before, during, and after the visits if the interactions with his mother were deemed to be contributory to his symptoms; and (c) for integrated intervention to take place, to find strategies to communicate a common understanding of the symptoms to Brandon’s caregivers, the child protection workers, and the court.

#### Intervention

In this case, the social workers were very sensitive to the child’s distress and needs; however, a psychological assessment was required for the Court to support their observations and, more important, to provide recommendations for adjusting visiting modalities in accordance with the child’s needs.

The psychological assessment included two assessments of Brandon’s attachment with his grandmother at a 1-year interval, a review of the observational data gathered by the grandmother, the observations of mother–child interactions, and information about Brandon’s general adaptation as provided by questionnaires filled out by the grandmother and the daycare worker. A final observation segment was done with Brandon, his grandmother, and his mother. This was conducted as part of the assessment and further served the purpose of demonstrating to the mother that using the grandmother as a secure base could gradually facilitate Brandon interacting with his mother within only a few minutes, even if he initially hid from her and stopped talking. Following this visit, the mother seemed to understand that taking into account her son’s need for security could facilitate the development of a positive relationship between them.

#### Outcome

After reviewing the evidence submitted, the Superior Court judge ruled in favor of a maximum of 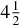 hr of visits per week with Brandon and his mother instead of leaving the Youth Protection Services solely to determine the modalities. Of note, the ruling also indicated that visits could be decreased or even canceled if Brandon showed too much anxiety. In the meantime, the mother experienced visiting her son at his grandmother’s home and was glad to realize how this setting was beneficial for her son and their relationship. Most visits now take place once or twice a week at the grandmother’s home, a secure base environment for Brandon, who is now 42 months. With the increased attunement of the various people responsible for Brandon’s sense of security—the Court, the workers, and the now-collaborating caregivers—the visits have evolved to allow Brandon to show significantly less anxiety and more and more pleasure when spending time with his mother.

From an attachment perspective, the reactions described earlier are linked to the child experiencing the perceived threat of the loss of his grandmother, who had become his main attachment figure, while he visited alone with his mother. Even if the biological parent’s behavior during the visit was not necessarily problematic, per se, reactions were usually observed when the child returned to his attachment figure, where he showed several anxiety reactions such as clinging and sleeping difficulties. Given the severity of his symptoms, it also is possible that Brandon was reexperiencing preverbal traumatic memories at a physiological level (Berger & Rigaud, 2001) since he had been initially removed from his parents due to neglect. The aggressive reactions were directed toward his grandmother, the attachment figure, because he held her responsible for his pain and distress. It was imperative that decision makers understood the child’s experience of being sent alone to meet a familiar figure that nevertheless could not provide security for him and respond accordingly. Such persisting intense reactions need to be considered seriously because in the long-term they can compromise the child’s development, jeopardize the placement, and increase the risk for psychopathology. Also of importance, these reactions do not favor the establishment of a positive relationship with the biological parent as so strongly desired, but lead on the contrary to the biological parent being perceived more and more as threatening for the child.

#### Alternative explanations of child’s symptoms

When dealing with the courts and even sometimes within the Youth Protection Services, alternative explanations of the child’s symptoms can be presented. The problems may be attributed to the foster family being in competition with the biological family and therefore wanting them out of the picture, which can lead to loyalty conflict for the child. Sometimes workers even conclude that the foster parents cannot properly comfort the child instead of considering that the child is bearing too much stress. Of course, both explanations can hold truth, and our job as infant mental health practitioners is to provide balanced recommendations to the courts with the goal of supporting decisions made from the child’s perspective, which is, in our opinion, too often understated.

### Cross-Cultural Practice Applications: New Developments in Scotland

We turn to Scotland to illustrate how infant mental health practitioners are sharing knowledge and practice expertise internationally to influence child welfare systems and the research base about young children in out-of-home care.

Maltreatment is the main reason for removing children under 5 years of age from the care of their parents in Scotland. Assessment of the quality of infant–parent relationships therefore has an important part to play in shaping the decisions about the future care of the child. We already have indicated the relative shortage of empirical studies which have shed light on this important stage in the childcare process. We now discuss one particular model of infant mental health practice which offers a systematic approach in this area and consider some practical implications associated with its implementation.

#### The Tulane Infant Team model

Zeanah and colleagues, working with families of children under the age of 5 years who are in foster care because of maltreatment, developed a model of intervention (Zeanah et al., 2001; Zeanah & Larrieu, 1998) which appears to address a number of the issues under consideration in this article. First, their model of intervention has a central focus on the relationship between the child and the caregiver (both biological and foster; Larrieu & Bellow, 2004). This is evident during both assessment and treatment. In the assessment phase, each significant caregiving dyad is studied using the Crowell procedure (Crowell & Feldman, 1988), an exercise involving tasks of increasing developmental difficulty and a short separation–reunion episode. Each of the caregiving adults also takes part in a Working Model of the Child Interview (Benoit, Zeanah, Parker, Nicholson, & Coolbear, 1997), which investigates the adult’s own attachment status and his or her view of the child.

The focus on the relationship also is evident in the treatment phase. Thus, a second component is that every family referred to the Tulane Team is given the opportunity to take part in treatment aimed at improving parent–child relationships while children are in care. This usually takes the form of a dyadic therapeutic intervention, often Infant–Parent Psychotherapy (Lieberman, Horn, & Ippen, 2005), but the specific choice of intervention is tailored to the individual case. This work is based on the premise that wherever it can be shown to be in the child’s best interest, the child should be reunified to the care of the parent. In addition, the Team offers intervention for children and their foster parents when needed to ensure stability and security for the child while in care (for a description of this intervention, see Heller, Smyke, & Boris, 2002, and Zeanah & Smyke, 2005).

Finally, the Tulane model is centered on a close working relationship with both social services and the legal system (Smyke, Wajda-Johnston, & Zeanah, 2004). Note that while social services and mental health services offer advice and recommendations about the contact arrangements and the long-term care of children, the decisions are made within a legal setting. The Tulane Team provides a report to the legal system which offers an account of their assessment of the child–parent relationship to the judge who will decide on contact and present and future care of the child. The availability of such systematically derived and well-evidenced advice is a key element in this model.

Zeanah et al. (2001) reported that following their intervention, fewer children were likely to be returned to their birth parents than were a control cohort (34.7 vs. 49%). Where reunification did take place, the risk of a further episode of maltreatment was significantly reduced in the intervention group compared to that of the control group. In relation to the index child, the “relative risk reduction” was 52.4% while the risk of an index parent being involved in a subsequent incident of maltreatment with another child was reduced by 63.1%.

This model offers one way of ensuring that these difficult legal decisions are supported by information based on current evidence about child development and attachment. But, as Zeanah et al. (2001) noted, further investigation of the effectiveness of the intervention and its potential for application in other jurisdictions are required.

#### Case vignette

Clinical researchers in Scotland are involved in a major study of the implementation of the Tulane model in Glasgow. In the course of the development phase of this project (described in Minnis, Bryce, Phin, & Wilson, 2010), a pilot case quickly encountered a major dilemma about the relationship between the contact process and the parent–child assessment process.

#### Background

Two siblings (Jake, aged 18 months, and Melanie, 30 months) were living with foster parents because of concerns that they had been seriously neglected while in the care of their birth parents. Both children had initially shown significant developmental delay, particularly in relation to language, while Melanie’s social behavior was indiscriminate and inappropriate, and their interaction was very concerning. However, after some months with foster parents, their presentations had improved substantially. Contact with birth parents had not taken place since the move to foster care but was arranged to allow assessment of the quality of relationships between the children and their parents. This exercise was immediately associated with a number of difficulties; for example, the birth parents engaged in a heated argument while the children were present. However, the more concerning development was what happened with Jake and Melanie in the immediate aftermath. They both became distressed, aggressive, and behaved in such a problematic fashion that their experienced foster parents soon began to doubt their ability to continue to care for the children.

#### Practice dilemmas

This case vignette raised a series of questions for the clinical researchers: First, should the assessment continue? Second, might an assessment process that required parental contact prove harmful to the children? Finally, would continuing the assessment endanger the stability of the foster placement, where both children had begun to make good developmental progress?

#### Considering the dilemmas

Within the Tulane Infant Team model, treatment is offered to every family where the court judges that maltreatment has occurred. The Tulane practitioners are therefore working routinely with children and parents whose relationship has been formally judged as harmful to the child. But in this pilot case, the researchers immediately faced a situation where the child welfare workers were so concerned about the distress shown by the children that they were inclined to reduce or end contact, despite the risk that important decisions about the care of the children would then have to be made with more limited information about the quality of the infant–parent relationship.

Discussion with the Tulane Team led to a useful distinction between experiences which may be stressful and those which can be reasonably regarded as being harmful to the child(ren). In the event, there was consensus among the Glasgow practitioners that this case fell into the latter category and that contact should stop. The reports to social services and the legal system would then reflect that opinion.

#### Outcome

The social services team marshaled the evidence available from their own practice and from those elements of the assessment which had been completed within the pilot project and applied to the appropriate legal forum (the Children’s Hearing) for an end to contact. The application was successful, and within weeks, both children settled in the foster placement. Social services then began proceedings for a Permanence Order which would allow the children to remain in their placement in the long-term. Although that legal process had yet to conclude, one of the foster parents later confirmed that the placement was again settled and secure.

Such dilemmas about how to balance the child’s well-being with the need to gather good-quality information and evidence are amplified by the lack of systematic research in this area. In the absence of such evidence, even decision makers with a good understanding of child–parent relationships and their significance for the mental health of the child will find considerable difficulty in steering a course which addresses the child’s rights.

The availability of well-evidenced methods for intervening to improve the experience of children who come into foster care because of maltreatment as well as their outcome and life chances of children is important, both for those providing helping services to children and for those making legal decisions about contact with their parents and their overall care. The Glasgow project serves to emphasize the importance of further research in relation to models of practice, such as the Tulane model, which have the potential to serve these important purposes.

### Influencing the System: Consultation to Child Welfare Workers in Louisiana, USA

One of the key lessons from the experiences we have described in Australia, Canada, and Scotland is the difficulty many workers and legal systems have in acknowledging the child’s rights and experiences and ensuring that these take precedence over those of the parents. Systematic assessment appears to be a helpful way for workers to achieve this. We will now discuss an innovative project from Louisiana that has attempted to improve foster care workers’ ability to recognize and act on the child’s needs.

From February 2008 to September 2010, foster care workers in Southwest Louisiana received training and consultation from experts in infant mental health regarding their cases involving children, ages 5 years and under, who were in foster care due to abuse or neglect. One of the goals of the state-funded program was to enhance worker effectiveness in identifying and responding to emotional, developmental, and attachment needs of young children in foster care. The consultation approach was developed from work of the Tulane Infant Team (Smyke et al., 2004; Zeanah & Larrieu, 1998; Zeanah et al., 2001; Zeanah & Smyke, 2005) and emphasized a “child-centered” model of foster care. In this model, the job of foster parents is to provide food, clothing, and shelter as well as love and attention, as if the child was their own biological child. They create a sense of *psychological safety* as well as provide physical safety for the child. The foster parent must become a secure base for the child while birth parents work to remedy the circumstances under which the child entered care (Heller, Smyke, & Boris, 2002; Smyke & Breidenstine, 2009).

The infant mental health consultation program was comprised of three components: *initial training*, *focus groups*, and *consultation*. Initial training consisted of 20 hr of seminar-format training in infant mental health and infant development, attachment, and caregiving relationships, and cultural issues given to foster care workers in early 2008. Focus groups with foster care workers and supervisors were conducted immediately following the third day of training to assess foster care workers’ needs related to providing service to young children in foster care. Following the initial training and focus groups, units of foster care supervisors and their workers began receiving weekly consultation by telephone or by videoconference. Consultants were faculty at a private medical school in Louisiana who have expertise in infant mental health and a great deal of experience working with the child welfare system.

Initially, each infant mental health consultant met with her assigned team of a supervisor and worker at their workplace. Following this, the consultant spoke with the supervisor and worker for 1.5 hr per week by telephone or occasionally by videoconference. The purpose of the consultation sessions were to assist workers and supervisors to “reflect” on the young children in their caseloads and to learn to apply state-of-the-art methods of infant and young child assessment, with a particular focus on caregiving relationships—including relationships with both birth parents and foster parents. This was an effort to apply and extend what was learned in the initial training to real-life situations faced by the workers and supervisors.

During consultation meetings, workers and supervisors were encouraged by consultants to discuss current cases from the perspective of caregiving relationships. Each participant had the opportunity to present and discuss the cases. Topics addressed during the initial training, including attachment relationships and early childhood development, were used as a framework for case discussions. The objective was that with increased worker knowledge and skills pertaining to enhancing caregiving relationships, workers would be better able to identify and address key issues in both foster and biological relationships.

The role of the infant mental health consultant was to provide support, information, and potential solutions to infant mental health concerns to foster care staff. The consultation process was intended to be case-specific, collaborative, voluntary, and confidential; that is, content was not discussed with upper level management. Foster care staff and consultants were expected to contribute equally during discussions.

Specific issues discussed included, but were not limited to, the following: identifying special needs of young children, including developmental and relationship needs with birth parents; enhancing caregiving relationships between foster parents and children; visitation between birth parents and their children; and transitioning children from foster homes to birth parent homes.

#### Case example

One of the most frequently discussed topics was “visitation with birth parents.” The remaining section is a description of an actual case (de-identified) presented by one worker about structuring visits.

#### Background

Over the course of three meetings, a relatively inexperienced worker presented the case of “James,” an 18-month-old male who had been in foster care for approximately 10 months due to his parents’ substanceabuse issues. By the worker’s report, the child was doing well in his foster home, and the foster mother had expressed interest in adopting him. The worker was very motivated to have the child reunify with his mother, even though she continued to use substances and was failing to complete the elements of her reunification case plan.

Each week, James traveled in a van with only the department’s transportation provider (a stranger to the child) for an hour to visit with his mother. Following this visit, he traveled another hour to visit with his father, before making the 2-hr trip back to the foster care office, where his foster mother picked him up. At times, the child traveled to the meeting place only to find that the birth parent missed the visit.

#### Practice dilemmas

As a starting point for discussion during the first consultation meeting regarding this case, the consultant (a) asked the team of foster care workers and their supervisor to consider what this ride must be like for the child. The team actively participated in this discussion, stating that the child might feel “scared” with no known adult traveling with him. The consultant (b) then asked the workers how much support they thought the birth parents should receive from their agency if they were not working on their case plans and were actively using substances. The consultant (c) inquired why the parents were not being asked to make the 1- to 2-hr drive to visit with their son. The supervisor stated that it was the policy of the agency to provide transportation to parents if they could not transport themselves. The consultant suggested that the parents demonstrate their willingness to sacrifice and see things from the perspective of their child to demonstrate commitment to reunification.

#### Practice dilemmas

The consultant made the following recommendations: (a) The birth parents needed to prioritize addressing the issues that brought their child into care before focusing on strengthening the relationships with their child. The rationale for this recommendation was to ensure that the neglect that brought the child into care would not recur, should the child be reunified. The consultant recommended that visits be reduced to the minimum allowed by the department’s policy (twice per month) until and if the parents engaged in substance-abuse treatment; then the visits may increase. (b) The parents should demonstrate a commitment to reunifying with their child by getting themselves to at least one office visit per month. The parents should call to confirm the visit within a reasonable amount of time. If the parents could not transport themselves, the agency would pick them up and bring them to the office rather than having the 18-month-old travel in the van to meet them. (c) On alternating visit weeks, the child should be accompanied by his foster mother (and primary attachment figure) for the long ride. If the foster mother could not attend the visit, the worker should accompany the child, as this child knew the worker and felt comfortable with her. The workers agreed to the recommendations and implemented this plan.

#### Outcome

Unfortunately, the birth parents were not able to remain clean and sober; thus, the case plan was changed from reunification to adoption, and the Court approved this change. Although the worker in this case expressed disappointment that the birth parents would not be able to raise their child, the consultant encouraged her and her team to view the outcome from the perspective of the child’s need for stability and security.

## DISCUSSION

Although child welfare law in the United States emphasizes the best interest of the child in permanency planning, the observations made by consultants involved with this program were that the child welfare system struggled with how to consider the immediate needs of the child residing in foster care, for example, when developing case plans and making day-to-day decisions such as determining the frequency, length, and structure of visits with birth parents. This struggle was reflective of the limited knowledge and understanding of what young children in foster care need for optimal development and mental health. Another explanation may be that foster care policy often does not specifically address the needs of children ages 5 years and under. For example, foster care policy in Louisiana states that parent visits shall occur at least every 2 weeks unless case circumstances prevent visiting or indicate otherwise. Further, in the first 6 months of placement, efforts are made to hold visits more often and to increase the length of visits. However, this protocol does not take into consideration the age of the child. From an attachment perspective, it would not be essential for the 1-month-old child of a parent who is actively using substances to visit with the mother more than twice per month, given that the focus should be on ensuring a secure attachment relationship develops with the child’s foster mother, who will provide comfort, security, and safety while the birth mother remedies the circumstances under which the child entered care.

In this consultation program, it was observed that foster care workers ultimately turned to agency policy when making decisions about the children in their caseload, especially when there was a disagreement among staff about how to approach a case. Data are currently being analyzed to assess the outcomes of the infant mental health consultation provided to foster care workers in one region of Louisiana. Future programs focused on enhancing foster care for young children might be improved by providing further training and consultation to upper management and the Courts, as the final decision-making authorities. An ultimate goal is to tailor child welfare policy to the appropriate developmental and attachment needs of young children, whose needs are unequivocally different from those of older children.

## CONCLUSIONS

Planning for contact between young children in foster care and their biological parents poses difficult dilemmas, but certain principles prevail. If the child is removed from his or her biological family at a very young age (e.g., <5 years), practitioners need to ensure the security of the infant’s primary caregiving relationship (foster parent or kinship caregiver) while also supporting the existing or developing attachment tie with the biological parent(s) where possible as parental capacities are assessed. The experiences from all four countries demonstrate the necessity of early and ongoing decision making to optimize the child’s development and well-being while he or she is in care. A child’s negative reactions to visits with biological parents may be due in large part to his or her developing a secure attachment to the foster family and should influence the decision process. Once a decision is made toward long-term placement or adoption, for certain children, occasional visits (three to four times a year) may be maintained as an “acquaintance link,” but only if child does not react negatively. These visits need to be guided by clear and realistic goals and offer the presence of a significant accompanying person (including during transportation). With infants and younger children, the presence of the foster parent therefore can be mandatory. The frequency and duration of visits need to be limited and adjusted to the careful observations of child’s reactions before, during, and after visits, flexibly adjusting modalities according to child’s reactions while paying special attention to emotional withdrawal, which is often more subtle. Despite all these precautions, visits sometimes have to be suspended or ended due to persistent distress and emotional harm to the child.

While innovative programs serving infants in out-of-home care exist, such as the Attachment and Biobehavioral Catch-Up intervention with foster parents (Dozier, Bick, & Bernard, 2011; Dozier et al., 2009), further research is needed to determine best practices for facilitating contact between these infants and their birth parents. The authors of the recent *JCCP* article (Leve et al.,) reviewed eight evidence-based interventions that promote resilience for foster children. Only three of these eight interventions were specifically geared toward young children, and only one intervention targeting young children, the Multidimensional Treatment Foster Care for Preschoolers (see Leve et al., 2012), includes a component which addresses the needs of the biological family. Nevertheless, the *JCCP* article has suggested that there is a growing empirical base that demonstrates the negative impacts of placement disruption and programmatic and policy-driven attempts to facilitate placement stability, notably (but not exclusively) through attachment-based interventions. The current article illustrates international efforts which take the infant’s perspective at the clinical practice, program implementation and evaluation, and child welfare system levels. Although we may judge the benefit of the practices described to the infant in the here and now by assessing their behavior and reactions, the long-term outcomes of systems operating from the infant’s perspective warrant further study. Further, controlled studies are needed to investigate the impact of enhancing the quality of visits on the emotional functioning of the child(ren), on the foster parent’s psychological commitment to the child(ren) in their care, and on the birth parents’ commitment to reunifying with their child(ren).

Finally, we believe that to have a more infant-informed system, child welfare workers and court personnel need training and consultation regarding manifestations and development of attachment (with biological and psychological parents), symptomatic manifestations in children (separation anxiety, traumatic stress reactions), and deleterious (and potentially irreversible) effects of repeated threats to attachment relationships. This training may take the form of didactic education to large numbers of staff and/or consultation on a case-by-case basis. In addition, children and families may need evaluation and treatment from practitioners well-versed in infant mental health, and child welfare workers need to be able to identify concerns and refer when needed. Above all, we think that despite pressures from biological parents, advocates of parents’ rights, or the judiciary, the child’s needs—especially those of “voiceless” and therefore vulnerable infants—must be prioritized.

## Footnotes

1 In Scotland, legal decisions about the welfare of children are dealt with through a unique system. While some specific matters of law are dealt with by the Court, most matters of welfare are remitted to a forum called the “Children’s Hearing.” This tribunal, consisting of three lay people with some basic training in child welfare and child law, is authorised to make key decisions such as frequency of contact and reunification with parents.
